# Phytohormone signaling pathway analysis method for comparing hormone responses in plant-pest interactions

**DOI:** 10.1186/1756-0500-5-392

**Published:** 2012-07-31

**Authors:** Matthew E Studham, Gustavo C MacIntosh

**Affiliations:** 1 Bioinformatics and Computational Biology Program, Iowa State University, Ames, IA, 50011, USA; 2 Department of Biochemistry, Biophysics, and Molecular Biology, Iowa State University, Ames, IA, 50011, USA

## Abstract

**Background:**

Phytohormones mediate plant defense responses to pests and pathogens. In particular, the hormones jasmonic acid, ethylene, salicylic acid, and abscisic acid have been shown to dictate and fine-tune defense responses, and identification of the phytohormone components of a particular defense response is commonly used to characterize it. Identification of phytohormone regulation is particularly important in transcriptome analyses. Currently there is no computational tool to determine the relative activity of these hormones that can be applied to transcriptome analyses in soybean.

**Findings:**

We developed a pathway analysis method that provides a broad measure of the activation or suppression of individual phytohormone pathways based on changes in transcript expression of pathway-related genes. The magnitude and significance of these changes are used to determine a pathway score for a phytohormone for a given comparison in a microarray experiment. Scores for individual hormones can then be compared to determine the dominant phytohormone in a given defense response. To validate this method, it was applied to publicly available data from previous microarray experiments that studied the response of soybean plants to Asian soybean rust and soybean cyst nematode. The results of the analyses for these experiments agreed with our current understanding of the role of phytohormones in these defense responses.

**Conclusions:**

This method is useful in providing a broad measure of the relative induction and suppression of soybean phytohormones during a defense response. This method could be used as part of microarray studies that include individual transcript analysis, gene set analysis, and other methods for a comprehensive defense response characterization.

## Findings

### Background

Plant hormones are involved in many aspects of plant development and responses to biotic and abiotic stresses. The three major phytohormones responsible for mediating defense responses to pests and pathogens are jasmonic acid (JA), ethylene (ET), and salicylic acid (SA) [1-3]. Recently, the participation of other hormones in defense signaling has become evident [3]. Among these, abscisic acid (ABA), a hormone normally associated with responses to abiotic stress, has been recognized as an important fine-tune regulator of defenses [4,5].

The production of these defense hormones is induced upon attack and it mediates a series of effective responses that can involve production of antibiotic compounds, production of volatiles emitted to attract predators of the attacker or discourage further attacks, programmed cell death to deprive the invader of nutrients, or other defensive changes depending on the type of pest or pathogen. Plant defense responses are often categorized based on the phytohormone able to trigger a specific response against the invader, although the existence of crosstalk between pathways is well known [1].

Decades of plant defense research has provided many examples of effective defense hormones for a multitude of plants. The oxylipin JA is the most prevalent defense hormone implicated in responses to insects and other invertebrate herbivores in Arabidopsis and other plants (reviewed in [6]). The phenolic SA is the most prevalent defense hormone in interactions with biotrophic pathogens and often induces the expression of pathogenesis-related (PR) proteins (reviewed in [7]). SA is also involved in gene-for-gene resistance, which includes a form of programmed cell death known as the hypersensitive response (HR). ET, best known for its role in fruit ripening, is also often induced as part of plant defenses, coordinating specific responses or participating in the modulation of JA- and SA-associated responses [8].

In addition to ET, JA, and SA, other hormones also participate in the coordination of defense responses [3]. Abscisic acid is a phytohormone predominantly involved in abiotic stress responses, but accumulating evidence shows that it is also active in defense (reviewed in [5]). ABA is normally considered a susceptibility determinant due to its role as negative regulator of disease resistance [4]; however, both positive and negative effects on defense responses have been reported for this hormone [5]. There are also many examples of interactions among these phytohormones (reviewed in[3], [9]). ET and JA work in concert to enhance defenses in a phenomenon called induced systemic resistance (ISR) [10]; while SA and JA are normally considered antagonistic signals (reviewed in [11]), although synergistic interactions have also been documented [12]. The regulation of defense responses by ABA is complex and the divergent effects observed in different systems seem to indicate that ABA’s effect on other hormone pathways is specific to each plant-pathogen/pest interaction[5]; in any case, general negative effects have been reported on SA biosynthesis and SA-elicited defenses and on the JA/ET pathway [4,5].

Interestingly, pests have evolved mechanisms to take advantage of the hormone crosstalk that controls plant defenses [13]. Some virulent pathogens can produce plant hormones or hormone analogs, presumably manipulating plant signaling to induce an ineffective decoy response that suppresses effective defenses. The most well-studied hormone mimicry strategy is the production of coronatine, an active analog of JA, by some strains of *Pseudomonas syringae*. Bacteria-produced coronatine induces a JA response that inhibits SA signaling and SA-mediated defenses [14], which are the effective defense against *P. syringae*[15-17]. Some *P. syringae* strains that infect soybean can produce ET, and bacteria deficient in this pathway are less virulent [18], probably because ET can also interfere with the production of an effective SA response.

Direct measurement of hormone levels is the most straightforward method of studying hormone induction, however it does not indicate if the signaling events triggered by hormone accumulation are active. Marker genes, whose transcripts are mainly induced by only one hormone, have been very useful in studying and verifying the induction of phytohormones. Examples include *ATAF1*[19] for ABA, ACC oxidase [20] for ET, *JAR1*[21] for JA, and *EDS1*[22] for SA. However, it is risky to base induction conclusions on one gene when hundreds are involved in each phytohormone signaling pathway. Moreover, crosstalk interactions between hormones could influence the expression of specific markers in ways not yet described.

The increase in genome-wide transcriptome analyses provides an excellent opportunity to determine the participation of each hormone in a particular plant-pathogen/pest interaction. An example of an existing phytohormone analysis tool is the “HORMONOMETER”, which can be used to determine hormone activity in transcriptome studies in Arabidopsis [23]. The HORMONOMETER uses the correlation between transcript profiles from hormone treatments and experimental studies to ultimately determine the effect of each experimental treatment on phytohormone pathways. This method relies on the large accumulation of publicly available transcriptome data for this plant.

Unfortunately, other plant systems are lagging behind, and not many transcriptome analyses of hormone responses are available in public databases. Moreover, annotation of individual genes and hormone pathways are not well developed. This is particularly evident for soybean, an important crop whose genome was recently sequenced [24]. Thus, for soybean and other plant systems with less extensive transcriptome analyses the HORMONOMETER approach cannot be implemented.

We analyzed the changes in the soybean transcriptome elicited by soybean aphid colonization [M.E. Studham and G.C. MacIntosh, submitted elsewhere]. Plant-aphid systems have the potential for the establishment of effective and decoy responses; thus, analysis of hormone pathways and signaling components is particularly important. To this purpose, we developed a comprehensive soybean pathway analysis that considers all soybean annotated genes associated with ABA, JA, ET, and SA, including receptors, regulators, biosynthesis genes, catabolism genes, and response genes. For each hormone, microarray results for the set of genes are used to produce a score that indicates how much the whole hormone pathway is affected by the pest. This score can then be used to compare different hormones and determine which hormones are more relevant in each given treatment and time point. To test our method we analyzed two well-described soybean defense responses for which microarray data were available in public databases.

## Method

### Pathway genes

Initially we referred to scientific literature reviews [7,25-29] to determine soybean genes associated with the ET, ABA, JA and SA pathways and split them into the following categories: biosynthesis, catabolism, receptors, regulators, signaling, and response. Annotations for probe sets in the Affymetrix GeneChip® Soybean Genome Array were downloaded from the Soybean Breeder’s Toolbox SoyChip Annotations (http://www.soybase.org). These annotations provided us with Arabidopsis homologs, Gene Ontology (GO, http://www.geneontology.org) biological processes, and homologs from other plant species. The Arabidopsis homologs and GO biological processes were used to identify hormone-associated genes. Below is a list of GO biological process names that were queried. The “<pathway>” word is a wildcard representing the pathway name (e.g. abscisic acid) and the pathway role is in parentheses:

“detection of < pathway > stimulus” (GO receptors)

“<pathway > biosynthetic process” (GO biosynthesis)

“<pathway > catabolic process” (GO catabolism)

“<pathway > metabolic signaling” (GO signaling)

“regulation of < pathway > mediated signaling” (GO regulator)

“response to < pathway > stimulus” (GO response)

After finding relevant genes from the literature review, soybean genes homologous to pathway-related Arabidopsis genes, and GO biological process annotations, total numbers of pathway-associated soybean genes were: ABA 231, ET 161, JA 210, SA 140 (see Additional file 1). Each gene was assigned a positive or negative correlation to account for catabolic enzymes and certain negative regulators.

### Role weights

The roles were weighted to put more emphasis on pathway genes with roles considered more important in determining pathway induction/suppression. Roles for genes assigned manually based on literature were weighted 50% more than roles for genes assigned based on GO annotations. The justification for this difference is that we assumed that the literature was more reliable than GO annotations. In addition, Response roles weights’ were increased because by definition response genes indicate that the hormone changes have resulted in a transcriptional response. The weights of Catabolism roles were decreased because although catabolism genes result in lower hormone levels, they also may be induced by the hormone. Therefore it is difficult to quantify the effect of transcript changes for catabolism genes in terms of hormone induction. Table1 lists all the role weights that were used for the pathway analysis.

**Table 1:** Weighting of different roles in hormone pathways

**Role**	**Weight**
Biosynthesis	1.5
Catabolism	1.0
Receptor	1.5
Regulator	1.5
Response	2.0
Signaling	1.5
GO Biosynthesis	1.0
GO Catabolism	0.5
GO Receptor	1.0
GO Regulator	1.0
GO Response	1.5
GO Signaling	1.0

Genes associated with phytohormones have different roles such as biosynthesis, response, regulation, and others. Each role was assigned a weight indicating its relative importance in determining the induction or suppression of the hormone. Roles with the “GO” prefix are for soybean genes that were included based on Gene Ontology biological process annotations and not initially found in the literature.

### Hormone pathway score

The pathway score indicates overall induction or suppression of a hormone for a given experiment and comparison, and it is calculated using microarray fold changes and q-values for pathway-associated transcripts. A single pathway score is calculated for each pathway and comparison (e.g. ABA in the day 1 susceptible response in soybean aphid experiment). The scores from different pathways can be compared to determine the predominant pathways in the comparison.

The first step in score determination is to filter out transcripts that do not meet differential expression criteria. For a given comparison, transcripts must have an absolute fold change ≥ 1.20 and a q-value ≤ 0.20 to be considered. We used cutoffs that are more relaxed than typical differential expression cutoffs for individual genes because significance and fold change are taken into account later in the scoring calculation and we did not want to ignore transcripts that barely missed the cutoffs. Next, the fold change values were converted to log_2_ differences to simplify the calculation.

Probe sets from the Affymetrix GeneChip® Soybean Genome Arrays do not necessarily have one-to-one relationships with soybean genes. In situations in which multiple probe sets corresponded to the same gene, the microarray results for all probe sets were averaged to obtain one value per gene. If a probe set was not assigned to any gene then it was treated as a separate unique gene. The reason for this conversion was to avoid over-weighting genes as a result of redundancy in the array.

A role score summarizing all the gene results in a particular pathway and role (e.g. ABA biosynthesis) is calculated by the following steps:

1. For genes that have a negative correlation, multiply the log_2_ difference times −1.

2. Transform the log_2_ differences based on significance (corresponding q-values):

a. For positive log_2_ differences: $diff*=diff+{\text{log}}_{2}\left(1\;–q\right);diff^{*}\geq 0$

b. For negative log_2_ differences: $diff*=diff–{\text{ log}}_{2}\left(1\;–q\right);diff^{*}\leq 0$

3. Sum up the transformed log_2_ differences and divide by the number of soybean genes to determine the role score. This score is now “per gene” to make it easier to compare scores among hormones that have differing degrees of signaling pathway elucidation.

The role scores are used to calculate the final pathway score for the comparison using the following steps:

1. For each role, divide the role weight by the sum of the weights of all represented roles to determine the adjusted role weight. This is necessary because not all roles have assigned genes and we don’t want to equate “lack of knowledge” with “no change”.

2. Multiply each role score times the adjusted role weight to determine the adjusted role score.

3. The pathway score for a comparison is the sum of the adjusted role scores multiplied times 100.

In addition to the scoring described above, we developed some variations such as absolute value scoring, experiment scores, role filtering, and gene marking scoring. These are described below:

*Absolute value scoring*: Use the absolute values of the fold changes from the microarray and always use a positive correlation.

*Experiment score*: Summarize the whole experiment by taking the average of the absolute values of the comparison scores.

*Role filtering*: Only consider certain roles (e.g. biosynthesis).

*Gene marker scoring*: Only consider one gene (e.g. ABA gene marker *ATAF1*).

In order to understand the scoring, consider a simple, unrealistic example in which all the genes (i.e. all probe sets) in a comparison showed a two-fold induction for a particular pathway. Also, the q-values for all the changes are near zero. In this example the pathway score = +100.

### Microarray data

The raw microarray data used to test our hormone analysis method were obtained from PLEXdb experiment “GM2” (http://www.plexdb.org/plex.php?database=Soybean) [30] and ArrayExpress experiment “E-MEXP-876” (http://www.ebi.ac.uk/arrayexpress). Normalization and statistical analyses were performed as described by M.E. Studham and G.C. MacIntosh (submitted elsewhere). Briefly, the Bioconductor [31] and affy packages [32] for the R programming language for statistical computing (version 2.6.2), were used throughout the statistical data analysis. Raw intensities were normalized using the GCRMA method [33,34]. For hypothesis testing, a moderated *t*-test [35] used a linear model to determine the p-values, which were then converted to q-values [36] to control the multiple testing error. The q-values enabled us to estimate the false discovery rate (FDR). Fold changes were calculated using the means of the normalized intensity values for the experimental and control treatments for each comparison.

## Results and discussion

Our pathway analysis method was applied to data for selected time points from two soybean defense response experiments: the response to Asian soybean rust (ASR, *Phakopsora pachyrhizi*) [37], and the response to soybean cyst nematode (SCN, *Heterodera glycines*) [38]. Both of these experiments used Affymetrix GeneChip Soybean Genome Arrays and both were subject to the same signal normalization and statistical analysis.

### Hormone signaling in response to ASR

**Figure 1:**
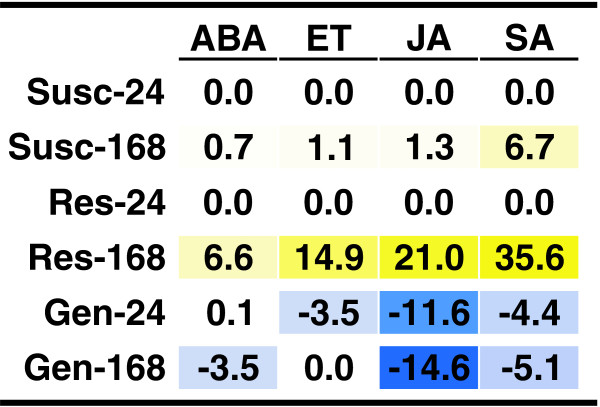
**Participation of phytohormones in the response to Asian soybean rust.** Hormone pathway scores indicating induction and suppression of signaling for the hormones abscisic acid (ABA), ethylene (ET), jasmonic acid (JA), and salicylic acid (SA) are shown for datasets corresponding to the susceptible (Susc) and the resistance response (Res) at 24 and 168 hours post-infection. The genetic differences (Gen) between susceptible and resistant plants were also analyzed. A three-color scale was used to highlight the scores: blue for negative (suppression), white for no change, and yellow for positive (induction).

According to van de Mortel *et al.*[37], soybean plants exhibited a biphasic response to ASR infection, which consisted of an initial response phase that is complete 24 hours after infection and a second response phase that begin at 72 hours in the rust-resistant plant and 96 hours in the rust-susceptible plant. For our analysis we only used the 24-hour and 168-hour time points from this experiment. We found that the susceptible and resistance response scores were zero at the 24-hour time point in the ASR experiment (Figure1). This is consistent with the negligible response to ASR reported by van de Mortel *et al.*[37].

At 168 hours, during the second phase, SA appeared to be the main regulator of ASR defense in both genotypes (Figure1, and Additional file 2). In the resistant response, ET and JA were induced at about half the SA induction level, while ABA was only weakly induced. In the susceptible response the only notable result was weak induction of SA (Figure1). In the original analysis of this dataset, van de Mortel *et al.* also concluded that the second phase of the response to this pathogen is faster and stronger in resistant plants (*Rpp2)* than in susceptible plants. While no direct conclusion was put forward on the role of hormones in these responses, genes regulated by SA and by JA were reported as differentially regulated in response to ASR [37]. Regulation of SA and JA related genes was also observed in an experiment that analyzed ASR-induced changes in gene expression in susceptible plants at different growth stages [39], and in work that used laser capture microdissection followed by microarray analyses to study the response of susceptible soybean palisade mesophyll cells infected with ASR [40]. Finally, the functional role of SA was confirmed by virus induced gene silencing experiments that targeted *GmNPR1*and other genes involved in SA signaling [41]. When these genes were silenced, *Rpp2*-mediated resistance was compromised.

Our analysis showed no hormonal action during the lull in the biphasic response to ASR and agreed with multiple rust studies indicating SA involvement in the defense response. These results show that our analysis is consistent with prior research regarding the soybean plant’s response to ASR. We also observed transcriptional differences between the mock-treated resistant and mock-treated susceptible plants that had not been reported previously (Figure1). Both JA and SA responses seem to be lower in resistant plants in uninfected conditions. Differences in gene expression between the two soybean varieties in control treatments are expected, since the varieties used were not near-isogenic lines.

### Hormone signaling in response to SCN

**Figure 2:**
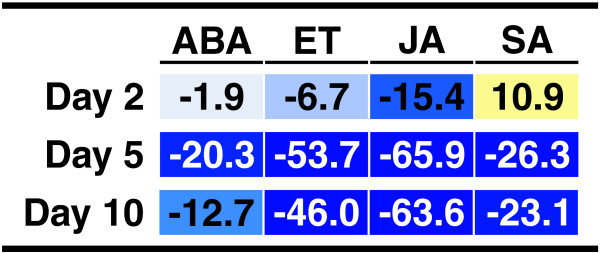
**Participation of phytohormones in the response to soybean cyst nematode.** Hormone pathway scores indicating induction and suppression of signaling for the hormones abscisic acid (ABA), ethylene (ET), jasmonic acid (JA), and salicylic acid (SA) are shown for datasets corresponding to the compatible interaction at 2, 5 and 10 days post-infection. The color scale is the same used in Figure1.

Ithal *et al.*[38] analyzed differences in gene expression between syncytial cells of SCN-infected soybean roots and uninfected root tissue at 2, 5, and 10 days post-inoculation. The hormone pathway analysis using our method on their data is shown in Figure2. We found SA induction and JA suppression on Day 2, then widespread suppression of phytohormone-related transcripts on Days 5 and 10. These results are consistent with the overall conclusion drawn by Ithal *et al.*, indicating that there is a general suppression of plant defense mechanisms by SCN during compatible interactions [38].

Several studies have proposed that ethylene participates in the establishment of successful root colonization by SCN. Arabidopsis mutants insensitive to ET show reduced root colonization by sugar beet cyst nematode than WT plants [42], and a similar phenotype was reported for soybean lines with reduced sensitivity to this hormone [43]. Thus, it was surprising to find a very strong suppression of the ET pathway in our analysis of the compatible interaction. However, several studies support our finding. In addition to the study by Ithal *et al.*, another microarray analysis found a general decrease in ET response in a compatible SCN-soybean interaction [44]. Moreover, direct measurement of ET levels indicated that ET accumulation is higher in uninfected than in SCN-colonized soybean roots [45].

Involvement of SA in soybean defense response against SCN has not been reported before. However, it is clear that SA is part of an effective defense response against sugar beet cyst nematode in Arabidopsis [46]. Moreover, an increase in SA levels has been observed in compatible and incompatible interactions between tomato and the potato cyst nematode, although SA response seems to be suppressed in susceptible plants [47]. Thus, the transient induction of the SA pathway in day 2 observed in our analysis could indicate a situation similar to that observed in tomato, where the plant initiates the establishment of an effective defense response early, but this response is suppressed by nematode colonization at later time points. Alternatively, the observed high score for the SA pathway could be the result of the induction of genes shared by the SA pathway and other response. Ithal *et al.*[38] observed the induction of genes related to cell wall rigidification. Upon closer inspection of our analysis (see Additional file 3), the score for SA on Day 2 was mostly due to the increased expression of phenylalanine ammonia-lyase 1 (*PAL1*) transcripts. *PAL1* is an SA biosynthesis gene but is also important in the lignin biosynthesis pathway. Since SA response and signaling genes were not induced, the *PAL1* induction could be directed towards lignification and cell wall rigidification, not SA biosynthesis. These hypotheses should be tested further to understand the role of SA in SCN-soybean interactions.

In conclusion, we developed a tool to analyze transcriptome data that is straightforward to apply to any dataset. The method was validated through the analysis of microarray data for well-characterized plant-pathogen interactions. The results obtained when we analyzed two sets of data corresponding to soybean interactions with SCN and ASR are in agreement with extensive research done in those two systems. Thus, this simple method should be useful to generate novel hypotheses and understand the participation of different phytohormones in different physiological processes. While our method was developed for soybean, it should be easily modified to use with any other plant system for which genomics resources are still limited.

## Abbreviations

ABA, Abscisic acid; ASR, Asian soybean rust; ET, Ethylene; GO, Gene Ontology; JA, Jasmonic acid; SA, Salicylic acid; SCN, Soybean cyst nematode.

## Competing interests

The authors declare that they have no competing interests.

## Authors’ contributions

MS designed, developed, and tested the method. GM conceived the study. MS and GM prepared the manuscript. All authors read and approved the final manuscript.

## Supplementary Material

Additional file 1 ** List of genes used to generate the hormone pathway scores.** This spreadsheet shows the genes used to generate the score for each hormone pathway and the ID of the Affymetrix chip probes corresponding to each gene. Click here for file

Additional file 2 ** Salicylic acid pathway activity in Asian soybean rust study.** This spreadsheet shows the changes in transcript accumulation for all SA-related genes from the day 1 and day 7 time points from the Asian soybean rust experiment. A summary also shows a breakdown of the pathway score into scores for each role. Click here for file

Additional file 3 ** Salicylic acid pathway activity in soybean cyst nematode study.** This spreadsheet shows the changes in transcript accumulation for all SA-related genes from all time points from the soybean cyst nematode experiment. A summary also shows a breakdown of the pathway score into scores for each role. Click here for file
